# Patterns and Predictors of Incident Return to HIV Care Among Traced, Disengaged Patients in Zambia: Analysis of a Prospective Cohort

**DOI:** 10.1097/QAI.0000000000002554

**Published:** 2020-11-04

**Authors:** Laura K. Beres, Sheree Schwartz, Sandra Simbeza, John McGready, Ingrid Eshun-Wilson, Chanda Mwamba, Kombatende Sikombe, Stephanie M. Topp, Paul Somwe, Aaloke Mody, Njekwa Mukamba, Peter D. Ehrenkranz, Nancy Padian, Jake Pry, Carolyn Bolton Moore, Charles B. Holmes, Izukanji Sikazwe, Julie A. Denison, Elvin Geng

**Affiliations:** a Department of International Health, Johns Hopkins Bloomberg School of Public Health, Baltimore, MD;; b Department of Epidemiology, Johns Hopkins Bloomberg School of Public Health, Baltimore, MD;; c Centre for Infectious Disease Research in Zambia, Lusaka, Zambia;; d Department of Biostatistics, Johns Hopkins Bloomberg School of Public Health, Baltimore, MD;; e Division of Infectious Diseases, Washington University School of Medicine, University of Washington, St. Louis, St. Louis, MO;; f College of Public Health, Medical and Veterinary Sciences, James Cook University, Townsville, Australia;; g The Bill & Melinda Gates Foundation, Seattle, WA;; h Division of Infectious Diseases, University of Alabama at Birmingham, Birmingham, AL;; i Division of Epidemiology, University of California Berkeley, Berkeley, CA; and; j Department of Medicine, Georgetown University, Washington, DC.

**Keywords:** HIV, Zambia, retention, antiretroviral therapy

## Abstract

Dynamic movement of patients in and out of HIV care is prevalent, but there is limited information on patterns of patient re-engagement or predictors of return to guide HIV programs to better support patient engagement.

**Methods:**

From a probability-based sample of lost to follow-up, adult patients traced by peer educators from 31 Zambian health facilities, we prospectively followed disengaged HIV patients for return clinic visits. We estimated the cumulative incidence of return and the time to return using Kaplan–Meier methods. We used univariate and multivariable Cox proportional hazards regression to conduct a risk factor analysis identifying predictors of incident return across a social ecological framework.

**Results:**

Of the 556 disengaged patients, 73.0% [95% confidence interval (CI): 61.0 to 83.8] returned to HIV care. The median follow-up time from disengagement was 32.3 months (interquartile range: 23.6–38.9). The rate of return decreased with time postdisengagement. Independent predictors of incident return included a previous gap in care [adjusted Hazard Ratio (aHR): 1.95, 95% CI: 1.23 to 3.09] and confronting a stigmatizer once in the past year (aHR: 2.14, 95% CI: 1.25 to 3.65). Compared with a rural facility, patients were less likely to return if they sought care from an urban facility (aHR: 0.68, 95% CI: 0.48 to 0.96) or hospital (aHR: 0.52, 95% CI: 0.33 to 0.82).

**Conclusions:**

Interventions are needed to hasten re-engagement in HIV care. Early and differential interventions by time since disengagement may improve intervention effectiveness. Patients in urban and tertiary care settings may need additional support. Improving patient resilience, outreach after a care gap, and community stigma reduction may facilitate return. Future re-engagement research should include causal evaluation of identified factors.

## Abstract 2

Supplemental Digital Content is Available in the Text.

## INTRODUCTION

Re-engagement in care is a critical but poorly understood step in the HIV care cascade globally.^1–4^ Dynamic movement of patients in and out of care is prevalent,^2,4,5^ making care interruptions part of the natural history of HIV treatment.^4,6^ These interruptions put patients at risk of poor health outcomes^7–9^ and the onward transmission of HIV.^10–12^ They threaten achievement of the global 95-95-95 targets.^13^ However, return to care is a positive patient behavior that has the potential to improve treatment outcomes. Especially as the burden of undiagnosed disease continues to diminish and time on treatment for the average patient increases, understanding how quickly disengaged patients return to care, what factors facilitate return, and ways to encourage more rapid return represents an important scientific agenda with a potentially significant magnitude of effect and public health relevance.^14^

HIV policy, service delivery, and monitoring must recognize and account for dynamic patient movement in the HIV care cascade.^3,6,15,16^ However, most extant literature focuses on the traditional, linear steps, including testing, linkage, antiretroviral therapy (ART) initiation, and viral suppression.^17^ Much less is known about patient re-engagement after a care-seeking absence. To date, the few return-to-care studies have been primarily retrospective and examined demographic and clinical characteristics alone. These studies suggest that between one-third and one-half of patients with a gap in care have a return visit,^3,18^ whereas studies including patient tracing observe return ranging from 20% to 70%.^19–21^ Factors associated with return in studies from East and Southern Africa include older age,^22^ lower CD4 count,^19,22^ female gender,^20,22^ health facility outreach,^19,20,22^ ART use,^19,22^ and latent patient factors related to poverty and poor care quality.^21^ Several additional factors were identified in a North American context, but not explored in studies conducted in African countries, including mental health concerns, secure housing, and substance use.^18^ Several qualitative studies have explored patients' experiences, identifying factors such as reduced stigma and social support as important for care engagement,^21,23–25^ but few studies measure these factors quantitatively to examine their association with return. There is a lack of prospective analyses of re-engagement that assess the effect of a comprehensive set of potential patient-related, clinical, and social influences on return.

To improve the understanding of re-engagement in HIV care and treatment in sub-Saharan Africa, our study prospectively identified incident return to HIV care and time to return among a representative sample of traced, lost to follow-up (LTFU) patients confirmed to be disengaged from care from 31 facilities across 4 provinces in Zambia. We conducted a risk factor analysis identifying predictors of return from a range of factors at the individual, social, and facility levels. This analysis can inform future research and intervention development through patient re-engagement risk stratification and hypothesis generation around re-engagement support opportunities.

## METHODS

### Study Background and Procedures

**FIGURE 1.**
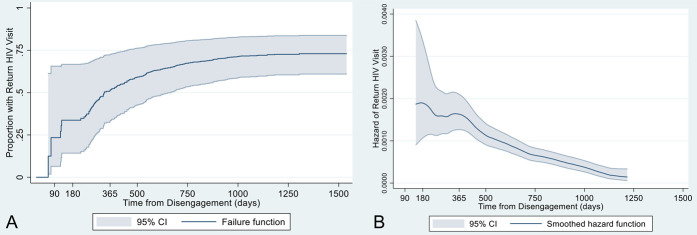
A, Cumulative incidence of re-engagement in care (n = 556). B, Hazard for returning to care based on time since disengagement.

This analysis is nested within a larger study, “Better Information for Health in Zambia” (BetterInfo).^26,27^ BetterInfo enumerated all LTFU adult patients at 31 sampled study facilities who had at least one HIV care visit between August 1, 2013, and July 31, 2015. Patients were determined to be LTFU if they were >90 days late for their last scheduled appointment and had a subsequent unknown care status. Approximately 10% of LTFU patients were then randomly sampled for BetterInfo study tracing. As described elsewhere,^26,27^ sampled patients were traced by a peer educator using paper medical record review, phone calls, and in-person visits to ascertain if the patient was (1) deceased, (2) alive and in-care, or (3) alive and out of care. All contacted, disengaged patients were verbally encouraged to return to care, and although not systematically applied, in some cases tracers accompanied the returning patient to the facility or met them for their return visit. On in-person patient contact, tracers obtained voluntary written informed consent and used tablet computers to administer a survey recording care status, demographic, social, behavioral, and household characteristics and reported barriers to care engagement. No medical care was administered during the tracing interaction. BetterInfo study surveys were administered in Nyanja, Bemba, Tonga, or English based on patient preference. Our nested study then extracted approximately 2 and a half years of follow-up HIV visit data after the cohort closed using electronic medical records (EMR) linked through unique patient identifiers (see Figure 1, Supplemental Digital Content, http://links.lww.com/QAI/B562).

### Study Population

Our analysis included all out-of-care patients identified through BetterInfo tracing who (1) confirmed that they did not have an HIV care visit since the last one identified in their medical record, (2) completed the study survey at the time of tracing, and (3) were interviewed after their estimated date of disengagement (>90 days from the last scheduled appointment based on the paper medical record review, as recorded in the study database).

### Measurements

Our study outcome, return to care, was obtained from facility visit dates in the EMR follow-up data extraction. Potential predictors of return (see Figure 2, Supplemental Digital Content, http://links.lww.com/QAI/B562) including clinical characteristics at the time of LTFU (eg, CD4 count, time in HIV care, and facility type) and gaps in care of >90 days before the BetterInfo study-identified gap were gathered from the patient's EMR at LTFU. All demographic (eg, age and marital status), social (eg, HIV status disclosure and stigma), behavioral (eg, alcohol use and travel), and household (eg, wealth and violence tolerance) factors potentially predictive of return were taken from the patient survey administered by the tracer. Missing survey items were taken from the EMR if available (eg, age and marital status). Most potential predictors were measured using closed-ended yes/no, multichoice, or Likert scale questions. To capture patient reasons for disengagement, changes needed to return, and return intentions, however, tracers asked the open-ended questions, “Why did you stop going to any clinic for HIV care?” and “What would have to happen for you to come back to care at any clinic?,” listened to the response, and recorded tick marks in as many predefined response options as were consistent with the patient's reply. Predefined subcategories developed through previous research^28^ included “structural” (eg, transport and work issues), “psychosocial” (eg, need encouragement, family, and disclosure issues), “clinic” (eg, poor care quality and wait too long), and “medical” (eg, felt well and too many pills) options, each of which had 4–13 detailed response options. The response category “other” captured responses that did not fit under the predefined options.

### Analysis

#### Potential Predictors of Return

Analysis of possible predictors of return to care was guided by an adapted social ecological conceptual framework^29^ of incident patient return to HIV care developed using extant literature^2,18–25,28,30–32^ and contextual knowledge (see Figure 2, Supplemental Digital Content, http://links.lww.com/QAI/B562). To model potential predictor variables, we first assessed the distribution of categorical variables, excluding variables where ≥97% of responses were the same. We assessed the relationship between continuous variables and return (on the log odds scale) using the LOWESS plots. Time from enrolment to disengagement was dichotomized at 18 months based on the LOWESS plot. From our 18 stigma questions developed to be consistent with draft and final HPTN 071^33^ stigma questions, we used exploratory and confirmatory factor analysis to identify 4 stigma subscales with adequate internal consistency: internalized (Cronbach α = 0.70), anticipated (Cronbach α = 0.87), experienced (Cronbach α = 0.72), and resilience (single question: “I confronted, challenged, or educated someone stigmatizing and/or discriminating against me”). Stigma subscale scores were summed from item responses. For internalized stigma, patient responses were dichotomized as low versus high at the median scale score. Experienced stigma was dichotomized as none versus any, and anticipated stigma was broken into approximate tertiles. Household wealth was estimated from ownership of 14 possible household items using the Demographic and Health Survey wealth index approach^34^ and broken into tertiles. Household violence tolerance scores were summed, with one point for each positive response to the 2, yes/no questions previously used in HIV research in Zambia, “If someone in the household misuses money is it acceptable to beat him/her?” and “In my household if a wife comes home late without permission of the husband, she will be beaten.”^35^ Alcohol use was analyzed using the AUDIT-C^36^ binge drinking question. For “patient reasons for disengagement” and “needs to return,” participants were analyzed as “yes” for a particular subcategory of “reason for stopping” or “need to return” if ≥1 detailed response option was selected for that patient under the specified subcategory. Subcategories were not mutually exclusive.^28^

We used descriptive statistics to assess missingness. If a participant was missing data on binge drinking but replied that they drank “≥5–6 drinks on a typical day” on a separate AUDIT-C question,^36^ their binge value was set to “yes.” For stigma subscales, we imputed the mean of available subscale items for a missing subscale item if at least 2 subscale items were available. We used multiple imputation with chained equations and 10 imputed data sets to account for remaining missing predictor data in the multivariable model.

#### Disengaged Patient Characteristics

We described the disengaged study population by potential predictors of return and used Kaplan–Meier methods to estimate the cumulative incidence of and time to return.

### Return to Care

We used Cox proportional hazards regression to estimate incident return to care. The time origin was the date of disengagement from care (90 days from the last appointment or 180 days from the last visit if the appointment date missing). The time scale was days since disengagement. Study entry was the date of in-person tracer contact, the point at which a patient was confirmed to be out of care. The event, incident return to care, is defined as the first HIV visit date of any type (ie, clinical, pharmacy, or laboratory) on or after the date of in-person tracing contact. Patients were censored at database closure.

We first examined the complete case, a univariate association of each potential predictor with return to care. The final multivariable model was informed by theory (see Figure 2, Supplemental Digital Content, http://links.lww.com/QAI/B562), including the following variables: gender, age, CD4 count at the last visit, time in HIV care, past care gaps, past facility outreach for return, facility type, mobility (having to travel for >1 month in the past year), and having a psychosocial reason for disengagement or psychosocial need to return. We additionally included factors with a univariate association significance of *P* < 0.05. We examined variance inflation factors to assess multicollinearity and examined Schoenfeld residuals and adjusted log–log plots for each covariate to assess the proportional hazards assumption.

### Supplemental Analyses

To better understand disengaged patients, we descriptively compared LTFU patients successfully traced and determined to be out of care with those found to be in-care.

To better understand return within a shorter time period, we conducted a supplemental analysis for incident return to HIV care within one year of disengagement, following the same analytic approach outlined above. To support a smaller model (more appropriate for fewer outcomes), the final supplemental multivariable model included only sex, age, and variables with a univariate association significance of *P* < 0.05. Acknowledging the important role of theory in a risk factor analysis, we also ran a multivariable model for return by one year with the theory-driven variables described above as a sensitivity analysis.

Analyses were conducted using Stata 15.1 IC (StataCorp, 2018) and Mplus 8.2 (Muthen & Muthen, 2018).

### Ethical Review

This study was approved by the University of Zambia Research Ethics Committee, the Zambian Ministry of Health, and the University of Alabama at Birmingham Institutional Review Board (UAB IRB). The Johns Hopkins University and the University of California at San Francisco had reliance agreements with the UAB IRB.

## RESULTS

### Disengaged Patient Characteristics

There were 556 patients identified through tracing as disengaged and included in our study sample (see Figure 3, Supplemental Digital Content, http://links.lww.com/QAI/B562). Disengaged traced patients were 41.7% men, had a median age at disengagement of 33.6 years [interquartile range (IQR): 28.4–39.9, min: 18.5, max: 80.3] and a median time in care before disengagement of 0.9 years (IQR: 0.4–2.6, min: 0.3, max: 10.7) (Table 1). The first supplemental analysis showed that, compared with LTFU patients successfully traced and determined to be in-care, disengaged traced patients were more likely to be men, younger, never married, to have had a higher CD4 count at the last visit, not yet initiated ART, and have been lost from a facility in the Lusaka Province (see Table 1, Supplemental Digital Content, http://links.lww.com/QAI/B562). Traditional healer contact was dropped from further analysis because of >97% of responses being the same.

**TABLE 1.** Disengaged Patient Characteristics (n = 556)

Potential predictors of return	Total	
	n	%
Sex		
Male	232	41.7
Female	324	58.3
Age at disengagement (yr)		
18–24	77	13.9
25–34	228	41.0
35–44	182	32.7
45+	69	12.4
Marital status		
Single or never married	115	20.7
Married	290	52.1
Separated, divorced, or widowed	151	27.2
Education		
No formal education	40	7.2
Primary	247	44.4
Secondary	218	39.2
Tertiary	51	9.2
Religion*		
Pentecostal	105	19.0
Universal Church of Zambia (UCZ)	37	6.7
Seventh-day Adventist	102	18.5
New Apostolic	79	14.3
Catholic	92	16.7
Other	137	24.8
Province		
Lusaka	238	42.8
Eastern	108	19.4
Southern	117	21.1
Western	93	16.7
Facility type		
Rural health center	131	23.6
Urban health center	301	54.1
Hospital	124	22.3
Last CD4 count (cells/µmol) before loss†		
<350	155	35.6
351-500	100	23.0
>500	180	41.4
Ill at enrolment, WHO stage III or IV or enrolment CD4 <200‡	162	30.9
Time from HIV care enrolment to disengagement		
≤18 mo	343	61.7
>18 mo	213	38.3
Previous gap in HIV care before study LTFU	203	36.5
Initiated ART	247	44.4
HIV status disclosure to someone	479	86.2
Patient ever contacted by facility in the past when missed a visit before study§		
No	472	86.1
Contacted as per standard of care (up to 3 times)	67	12.2
Contacted beyond standard of care (>3 times)	9	1.7
Travel time from usual residence to facility		
Less than 1 h	239	43.0
1 to under 2 h	139	25.0
2 hours or more	178	32.0
Did not spend >1 mo away from usual residence in the past year‖	255	46.7
Relationship to head of household¶		
Head	274	49.4
Wife or husband of head	155	27.9
Other	126	22.7
Used herbal remedies in the past 6 months#	29	5.3
No binge alcohol use**	367	67.8
Wealth tertile††		
Poorest	184	33.6
Middle	193	35.3
Richest	170	31.1
Tolerance of household violence‡‡		
No tolerance	426	80.2
Some tolerance	54	10.2
High tolerance	51	9.6
High internalized stigma vs. Low‖	218	39.9
Anticipated stigma††		
Anticipated, low	189	34.6
Anticipated, medium	144	26.3
Anticipated, high	214	39.1
Experienced stigma in the past 12 months#	133	24.5
Challenged, educated, or confronted stigmatizer in the past 12 months§§		
No	470	86.9
One time	29	5.3
More than once	42	7.8
Patient reported reasons for disengagement‖‖		
Any structural reason for stopping care	241	43.6
Any psychosocial reason for stopping care	227	41.1
Any clinic reason for stopping care	191	34.5
Any medical reason for stopping care	147	26.6
Patient reported needs for return to care¶¶		
Any structural barrier to return to care	96	17.7
Any psychosocial barrier to return to care	140	25.7
Any clinic barrier to return to care	229	42.1
Patient reported already planning to return	295	54.2

*n = 552, †n = 435, ‡n = 524, §n = 548, ‖n = 546, ¶n = 555, #n = 543, **n = 541, ††n = 547, ‡‡n = 531, §§n = 541, ‖‖n = 553—categories are not mutually exclusive, ¶¶n = 544—categories are not mutually exclusive.

### Patterns of Return to Care

Most disengaged traced patients, 73.0% (95% CI: 61.0 to 83.8) had a return HIV visit. The median follow-up time was 32.3 months (IQR: 23.6–38.9). The cumulative proportion of patients returning were 23.4% (95% CI: 6.5 to 65.7) by 90 days, 33.7% (95% CI: 14.2 to 66.7) by 180 days, and 51.4% (95% CI: 33.2 to 72.5) by 365 days (Figure 1A). The overall incidence rate of return is 0.73 per 1000 person years (95% CI: 0.64 to 0.84), declining with time since disengagement, and no additional returns after 3.5 years postdisengagement (Figure 1B). Among returners, the median time spent out of care was 19.1 months (IQR: 13.9–25.4).

### Predictors of Return to Care

Univariate analyses indicate that disengaged, traced patients were significantly (*P* value <0.05) more likely to return to care if they had been contacted more than 3 times by the facility after the past missed visits and if they had challenged, confronted, or educated someone stigmatizing them once in the past year. Patients were significantly less likely to return if they sought care from an urban health center or hospital, compared with a rural health center, or were from the richest wealth tertile (Table 2).

**TABLE 2.** Crude and Adjusted Predictors of Return to Care Among Disengaged Patients

Predictors of Return	Crude (Univariate, Complete Case Analysis)			Adjusted* (n = 556)		
	Hazard Ratio	95% CI	*P*	Hazard Ratio	95% CI	*P*
Male sex vs. female	1.02	0.77 to 1.35	0.90	0.91	0.65 to 1.26	0.57
Age at disengagement (yr)			0.55			0.65
18–24	1.00			1.00		
25–34	0.97	0.62 to 1.52		0.89	0.56 to 1.42	
35–44	1.16	0.74 to 1.82		1.01	0.63 to 1.64	
45+	1.28	0.74 to 2.20		1.21	0.68 to 2.17	
Marital status			0.64	//		
Single or never married	1.00					
Married	1.17	0.81 to 1.70				
Separated, divorced, or widowed	1.20	0.80 to 1.81				
Education			0.07	//		
No formal education	1.00					
Primary	1.29	0.73 to 2.25				
Secondary	0.95	0.54 to 1.69				
Tertiary	0.67	0.31 to 1.44				
Religion*			0.85	//		
Pentecostal	1.00					
Universal Church of Zambia (UCZ)	1.37	0.75 to 2.51				
Seventh-day Adventist	1.24	0.78 to 1.97				
New Apostolic	1.16	0.70 to 1.92				
Catholic	1.31	0.82 to 2.08				
Other	1.10	0.71 to 1.71				
Province			0.58	//		
Lusaka	1.00					
Eastern	0.96	0.65 to 1.42				
Southern	0.88	0.60 to 1.29				
Western	1.21	0.82 to 1.78				
Facility type			**<0.01**			**0.01**
Rural health center	1.00			1.00		
Urban health center	0.63	0.46 to 0.87		0.68	0.48 to 0.96	
Hospital	0.43	0.28 to 0.66		0.52	0.33 to 0.82	
Last CD4 count (cells/µmol) before loss†			0.38			0.63
<350	1.00			1.00		
351–500	0.75	0.49 to 1.15		0.85	0.55 to 1.32	
>500	0.85	0.59 to 1.20		0.83	0.55 to 1.25	
Ill at enrolment (WHO stage III or IV or enrolment CD4 <200) vs. not‡	1.23	0.90 to 1.67	0.19	//		
>18 months from HIV care enrolment to disengagement vs. ≤18 mo	0.96	0.72 to 1.29	0.81	0.58	0.36 to 0.94	0.03
Previous gap in care vs. no gap before study LTFU	1.25	0.94 to 1.66	0.13	1.95	1.23 to 3.09	**<0.01**
Initiated ART vs. no ART	0.88	0.66 to 1.16	0.36	//		
HIV status disclosure to someone vs. no disclosure	1.21	0.78 to 1.87	0.40	//		
Patient ever contacted by facility in the past when missed a visit before study§			**0.04**			**0.11**
No*	1.00			1.00		
Contacted as per the standard of care (up to 3 times)	0.91	0.58 to 1.44		1.09	0.68 to 1.75	
Contacted beyond the standard of care (>3 times)	2.84	1.26 to 6.43		2.65	1.04 to 6.73	
Travel time from usual residence to facility			0.32	//		
Less than 1 h	1.00					
1 to under 2 h	0.98	0.70 to 1.38				
2 hours or more	0.78	0.56 to 1.10				
Did not spend >1 mo away from usual residence in the past year vs. did‖	1.26	0.95 to 1.67	0.11	1.05	0.78 to 1.41	0.74
Relationship to head of household¶			0.57	//		
Head	1.00					
Wife or husband of head	1.16	0.83 to 1.62				
Other	1.17	0.83 to 1.65				
Used herbal remedies in the past 6 mo vs. did not#	1.38	0.77 to 2.48	0.28	//		
No binge alcohol use vs. binge alcohol use**	0.98	0.73 to 1.32	0.89	//		
Wealth tertile††			**<0.01**			**0.01**
Poorest	1.00			1.00		
Middle	1.17	0.84 to 1.62		1.27	0.89 to 1.80	
Richest	0.64	0.44 to 0.92		0.71	0.47 to 1.08	
Tolerance of household violence‡‡			0.75	//		
No tolerance	1.00					
Some tolerance	0.83	0.51 to 1.35				
High tolerance	0.99	0.59 to 1.66				
High internalised stigma vs. low‖	1.20	0.90 to 1.60	0.21	//		
Anticipated stigma††			0.40	//		
Anticipated, low	1.00					
Anticipated, medium	1.17	0.81 to 1.68				
Anticipated, high	1.26	0.90 to 1.76				
Experienced stigma vs. did not experience stigma in the past 12 months#	1.05	0.76 to 1.45	0.78	//		
Challenged, educated, or confronted stigamtizer in the past 12 months§§			**0.01**			**<0.01**
No	1.00			1.00		
One time	1.90	1.15 to 3.14		2.14	1.25 to 3.65	
More than once	0.63	0.33 to 1.19		0.65	0.33 to 1.27	
Patient reported reasons for disengagement‖‖						
Any structural reason for stopping care vs. no structural	1.05	0.79 to 1.39	0.74	//		
Any psychosocial reason for stopping care vs. no psychosocial	0.80	0.60 to 1.07	0.13	0.94	0.68 to 1.29	0.68
Any clinic reason for stopping care vs. no clinic	1.11	0.83 to 1.49	0.47	//		
Any medical reason for stopping care vs. no medical	1.13	0.83 to 0.45	0.52	//		
Patient reported needs for return to care¶¶						
Any structural barrier to return to care vs. no structural barrier	1.04	0.72 to 1.50	0.84	//		
Any psychosocial barrier to return to care vs. no psychosocial barrier	0.71	0.50 to 1.01	0.06	0.71	0.48 to 1.06	0.10
Any clinic barrier to return to care vs. no clinic barrier	1.07	0.80 to 1.42	0.66	//		
Patient reported already planning to return vs. not	1.05	0.79 to 1.40	0.72	//		

Entries in bold are significant at the <0.05-level or have a confidence interval not crossing 1.

*n = 552, †n = 435, ‡n = 524, §n = 548, ‖n = 546, ¶n = 555, #n = 543, **n = 541, ††n = 547, ‡‡n = 531, §§n = 541, ‖‖n = 553, ¶¶n = 544.

*Adjusted based on theory and 0.05 univariate significance: sex, age, last CD4 count, time since enrolment, past care gaps, past facility contact after loss, facility type, mobility, and psychosocial barriers to care.

Independent predictors of incident return to HIV care from the multivariable model with *P* values at or below 0.01 level included having had a previous gap in care (aHR: 1.95, 95% CI: 1.23 to 3.09) and the patient having challenged, educated, or confronted someone stigmatizing them once in the past year (aHR: 2.14, 95% CI: 1.25 to 3.65; more than once aHR: 0.65, 95% CI: 0.33 to 1.27). Patients were less likely to return to care if they sought care from an urban health center (aHR: 0.68, 95% CI: 0.48 to 0.96) or a hospital (aHR: 0.52, 95% CI: 0.33 to 0.82) compared with a rural health center (Table 2). Although the overall *P* value of the wealth tertile was 0.01, the hazard ratio estimates and confidence intervals did not show a consistent direction of association between increased wealth and return. (wealthiest aHR: 0.71, 95% CI: 0.47 to 1.08; middle tertile aHR: 1.27, 95% CI: 0.89 to 1.80) (Table 2).

### Supplemental Analysis: Predictors of Return by one Year

An estimated 51.4% (95% CI: 33.2 to 72.5) of participants returned by one-year postdisengagement. In the multivariable model built, based on significant predictors from univariate analyses, statistically significant (at the 0.01 level) independent predictors of incident return within 1 year of disengagement included being 45 years or older and having used herbal remedies in the past 6 months (see Table 2, Supplemental Digital Content, http://links.lww.com/QAI/B562). Patients were less likely to return by 1 year if they reported a psychosocial or clinic-related reason for stopping care (see Table 2, Supplemental Digital Content, http://links.lww.com/QAI/B562). The sensitivity analysis using the theory-driven model showed consistent results for the age and psychosocial reasons variables and identified no other significant predictors of return. Estimate precision was poor in these models due to limited events.

## DISCUSSION

With sufficient follow-up time, a high proportion of disengaged, traced patients, 73%, returned to care across 4 provinces in Zambia. More action needs to be taken, however, to hasten return. Among those patients returning to care, the median time spent disengaged was 19 months. Our data show that the rate of return is higher soon after disengagement. Earlier efforts to facilitate return may be more effective. Indeed, a retrospective analysis of patient outreach in Kenya demonstrated improved return with more rapid tracing.^22^ However, more rapid return soon after disengagement may also indicate that patients who do not return quickly may require targeted support to come back to care.

Interventions to support patient resilience to stigma and to limit stigma in the social environment may facilitate increased re-engagement. Our data indicate that, compared with not confronting stigma at all, confronting stigma once in the past year facilitates re-engagement. This is consistent with existing literature on the relationship between coping, resilience, and improved health outcomes.^37,38^ However, we do not see a traditional dose–response relationship because challenging stigmatizers multiple times does not further increase return. We theorize that the repeat confrontation of stigmatizers may represent a more hostile social environment or chronic stress, limiting any positive effect the ability to respond to a stigmatizer may bring. Research has shown that the effect of HIV stigma on health is worse in the context of low perceived community support^39^ and that the pathways through which resilience to stigma operates in the context of chronic stress are complex.^40^ Future re-engagement research should include stigma and resilience measures and test effectiveness of resilience interventions to improve return to care.^41,42^

Despite tracing, 27% of disengaged patients did not return to care by the end of study follow-up. Our data suggest that disengaged patients from urban health centers and hospitals are at a higher risk than rural patients of remaining disengaged and may require targeted interventions. Greater likelihood of return among those at rural health centers may be consistent with the more personal relationship-based care often available in rural, compared with urban and tertiary care centers. Existing research supports the importance of health care worker–patient relationships in patient engagement.^43,44^ In addition, urban versus rural patients may have different needs driving engagement. Past research has shown differences, for example, in which differentiated service delivery models for HIV treatment access are preferred between urban and rural patients.^45^ More research is needed to understand the mechanisms underlying facility-level difference in re-engagement and how to best address them to support return.

The finding that previous care gaps predict incident re-engagement adds additional urgency to the need to conceptualize care engagement as a dynamic process^4,5,10,14^ and the need for effective interventions to support continuity of care. Although complex factors are likely associated with both having a previous care gap and a patient's subsequent re-engagement, our findings suggest that investment in supporting patient return after one care gap may pay future re-engagement dividends. The greater than 2.5-fold increase in the hazard of return among disengaged patients who were repeatedly contacted by the clinic beyond the standard of care is consistent with this suggestion and other retention literature.^46,47^ Together these results warrant further investigation into the mechanisms through which extended outreach may support return, such as relationship development, and outreach effectiveness evaluation.

Our analysis suggests that factors predictive of return by one-year postdisengagement are more proximal to the patient care experience than predictors within the full study period. This suggests that effective interventions early on may need to target different mechanisms than interventions for people who remain disengaged for a longer time. In addition to older age and the use of herbal remedies in the 6 months before the survey, independent predictors of return by one year included not reporting a clinic-related complaint (eg, poor quality of care, lack of respect, and spending too much time at the facility) or a psychosocial reason (eg, clinic attendance creating conflicts, risking disclosure, being told to stop by someone influential, depression, and forgetting or seeking alternative care) for stopping care. Although self-treatment with herbal remedies may indicate illness-driven care seeking, finding ways to reduce clinic and psychosocial barriers, such as improving patient clinic experiences^43,44,48^ and engaging social support,^49,50^ may be important to encourage return sooner after disengagement.

## LIMITATIONS

Despite intensive tracing efforts, we were not able to obtain an updated vital or care status on 25% of the sampled patients. If disengaged patients not successfully traced are systematically different from those found, the estimates may be biased. By using EMR data to compare, patients we found were more likely to be from rural health centers and from provinces other than Lusaka, indicating that our estimates may over-represent rural experiences. These 2 groups were similar on other demographics (data not shown). Our study was only able to identify return among patients whose return care visit was documented using the same unique patient number in the 4 study provinces. It is possible that patients returned as a “new” patient under a new unique patient number or to a facility outside of the study area, potentially underestimating return. Patients in urban or tertiary care settings may have more health facility options due to higher facility density, which may make them more likely to have an undocumented return under a new patient number. Predictors were largely collected using survey responses, which are subject to self-report error, recall, and social desirability biases. As study observation began after disengagement, we assume that survey-measured predictors are time invariant in the interim. Because of the poor documentation of mortality in the EMR, we were unable to look at the competing risk of death.

## CONCLUSIONS

The most appropriate models of HIV care engagement show dynamic engagement patterns that demand multifaceted flexibility and support for retention, as is true for many chronic diseases.^51,52^ Return to care after disengagement is a critical yet under-researched step of the HIV care cascade. Our findings suggest that patients in urban and tertiary care settings may need additional return support and that efforts to improve patient resilience and outreach after any care gap may facilitate return. Other important re-engagement influences may include positive patient experience at the clinic, having a supportive psychosocial environment, not being in the wealthiest population tertile, and older age. Future re-engagement research should include measures of these predictors to investigate their mechanisms of effect and evaluate their causal effect on return to care.
